# Controlled Disassembly and Purification of Functional Viral Subassemblies Using Asymmetrical Flow Field-Flow Fractionation (AF4)

**DOI:** 10.3390/v10110579

**Published:** 2018-10-23

**Authors:** Katri Eskelin, Minna M. Poranen

**Affiliations:** Molecular and Integrative Biosciences Research Programme, Faculty of Biological and Environmental Sciences, Viikinkaari 9, University of Helsinki, FI-00014 Helsinki, Finland; minna.poranen@helsinki.fi

**Keywords:** enveloped virus, bacteriophage, virus disassembly, subviral particles, field-flow fractionation, size-based separation, dsRNA virus

## Abstract

Viruses protect their genomes by enclosing them into protein capsids that sometimes contain lipid bilayers that either reside above or below the protein layer. Controlled dissociation of virions provides important information on virion composition, interactions, and stoichiometry of virion components, as well as their possible role in virus life cycles. Dissociation of viruses can be achieved by using various chemicals, enzymatic treatments, and incubation conditions. Asymmetrical flow field-flow fractionation (AF4) is a gentle method where the separation is based on size. Here, we applied AF4 for controlled dissociation of enveloped bacteriophage φ6. Our results indicate that AF4 can be used to assay the efficiency of the dissociation process and to purify functional subviral particles.

## 1. Introduction

Viral genomes are protected by protein shells or lipid envelopes that also contain the needed information for host recognition. These protective layers may be relatively simple and contain only few virus-encoded protein components or may consist of complex combinations of protein shells and lipid bilayers [1,2]. Controlled dissociation studies provide information on the composition of virions, interactions between their components, stoichiometry of the subunits, as well as the possible role of different subparticles and virion components in the virus life cycle. Dissociation reactions are performed by combining various chemicals, enzymatic treatments, and incubation conditions.

**Figure 1:**
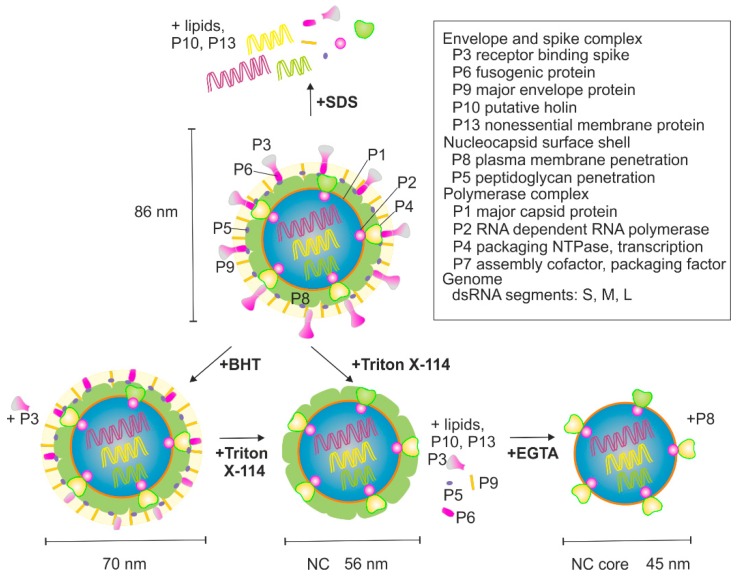
Schematic presentation of φ6 virion and the flow chart for the used treatments. An intact virion is shown at the top. The nucleocapsid (NC) core is made of four proteins P1, P2, P4, and P7 that encapsidate the three dsRNA segments L, M, and S. NC consists of the NC core that is covered with P8. The viral envelope embeds membrane proteins P6, P9, P10, P13, and P3 spike protein via P6. Treatments performed in this study with the expected outcome are also shown. See Materials and Methods for details.

φ6 virions are composed of protein (~70% of weight), lipid (~20% of weight), and double-stranded (ds)RNA (~10% of weight) [3,4]. The total mass of virions is ~99 MDa [4] and the reported diameter is ~70–86 nm, depending on the method used [4,5,6,7]. The viral genome is segmented into three dsRNAs molecules that are designated as small (S, 2949 bp), medium (M, 4065 bp), and large (L, 6374 bp), based on their different lengths. The genome encodes 14 proteins, of which 12 are found in mature virions (Figure 1) [8]. φ6 has served as a model virus to study how segmented RNA viruses package their genomes into virions [9,10,11,12], how RNA-dependent RNA polymerases function [13,14,15,16], how lipid-containing bacteriophages and their subcomponents are assembled and disassembled [6,17,18,19,20,21], and how the individual viral proteins or their subassemblies function as reviewed in [2].

The virion of φ6 has three biochemically distinct structural layers (Figure 1). The outermost layer is a host-derived phospholipid bilayer that embeds membrane proteins P6, P9, P10, and P13, whereas P3 spike protein is membrane-associated through its interaction with P6 [22,23]. The nucleocapsid (NC) resides underneath the envelope. It has two distinct proteinaceous layers (Figure 1). The outer surface of the NC is made up of the P8 layer that is interrupted with P4 hexamers protruding from the surface of the NC core [17,18,19]. The NC core contains four protein components: P1, P2, P4, and P7 [24]. The major surface component of the NC core is P1 which forms an icosahedral shell enclosing the genome. P4 is also attached to the surface of the P1 shell, whereas P2 and P7 reside inside [17,18,19,25,26].

Asymmetrical flow field-flow fractionation (AF4) is a chromatography-related method, where sample components are separated in a thin and narrow channel using the forces generated by two simultaneous flows: the channel flow and the cross flow. The channel flow has a parabolic profile. It transports the sample components towards the detectors and the fraction collector. Separation of sample components is achieved by applying an external field, the cross flow. The cross flow is perpendicular to the channel flow and it pushes the sample components towards the accumulation wall. This force is counteracted by the diffusion of molecules away from the accumulation wall. As a result, each sample component equilibrates at a distance from the accumulation wall that depends on their diffusion coefficient (D) and hydrodynamic size (R_h_) [27,28]. In the normal separation mode, which applies to sample components smaller than ~0.5 μm [29,30,31], larger sample components with lower diffusion coefficients equilibrate closer to the accumulation wall than smaller particles with higher diffusion coefficients. As a consequence, smaller molecules experience higher-velocity flows in the central region of the channel and elute first. In an optimal situation, AF4 can separate sample components with a broad size range in a single experiment (~10^3^–10^9^ Da, particle diameters from 2 nm to 0.5 μm) [31], provided that they have sufficiently different diffusion coefficients. For a detailed description of the AF4 principles and theory, several reviews and textbooks cover the subject well [29,32,33,34,35].

In this study, we performed various well established chemical and biophysical treatments for the dissociation of purified φ6 virions and showed that AF4 is a versatile and gentle method to study virus disassembly processes and for the isolation of biologically active viral subparticles.

## 2. Materials and Methods

### 2.1. Virus Purification and Treatments

φ6 was produced as previously described [5,36]. Purified virions were resuspended in a 10 mM potassium phosphate buffer (pH 7.2) supplemented with 1 mM MgCl_2_. Dissociation treatments, summarized in Figure 1, were performed as described earlier [36] with some modifications. P3 was removed by treating purified φ6 virions with 4 mM butylated hydroxytoluate (BHT) at 30 °C for 10 min. Total dissociation of particles was obtained by incubation at room temperature (RT) in the presence of 1% (*v*/*v*) sodium dodecyl sulfate (SDS).

NC was prepared by extracting the lipid envelope on ice by treating with 5% (*v*/*v*) Triton X-114 for 10 min. Triton X-114 was precondensed with 10 mM potassium phosphate (pH 7.2) and 150 mM NaCl [24] (Figure S1, Supplementary Materials). Presence of salt (NaCl/KCl) was needed for NC stability [24]. The NC-containing water phase was separated from the detergent phase by incubation at 30 °C for 5 min, followed by a slow-speed centrifugation at 1300× *g* for 5 min. The resulting sample was named “NC1 input” (Figure S1, Supplementary Materials). Alternatively, after extraction on ice, the preparation was layered on top of an 8% sucrose cushion (8% (*w*/*v*) sucrose, 10 mM potassium phosphate buffer (pH 7.2), 1 mM MgCl_2_, 0.1 mM CaCl_2_, and 150 mM NaCl) that was incubated at 30 °C for 5 min prior to centrifugation (Figure S1, Supplementary Materials). The obtained NC-containing water phase was named “NC2 input”.

P8 dissociation from the NC was triggered by Ca^2+^ chelation (Figure 1). NC2 input or the AF4-purified NC was used as a starting material. Uncoating was performed by treating the NC with 25 mM EGTA at RT for 30 min [24]. Uncoating reactions were supplemented with 1 mM ATP to stabilize the otherwise fragile NC core particles [37].

### 2.2. AF4 Instrumentation and Operation

The AF4 experiments and data collection were performed using an AF2000 MT instrument and Postnova AF2000 software (Postnova Analytics, Landsberg, Germany) as previously described [5,38,39], except that a teflon spacer of 250 μm and a regenerated cellulose (RC) membrane with a molecular weight cut-off (MWCO) value of 10 kDa (Postnova) were used (Lots 1551623 and 1653893). Channel flow was monitored in volts (V) at 260 nm (Shimadzu SPD-20A; Shimadzu, Kyoto, Japan). The AF4 channel temperature was 22 °C. The sample was injected during the focusing step using two opposing lateral flows (each 0.2 mL/min). Focusing (5 or 10 min) was followed by one min transition to the elution phase. During elution, a 5 min constant cross flow was applied prior to a 25 min linear cross-flow gradient from 1 mL/min to 0.1 mL/min, unless otherwise mentioned. Final elution was done using a constant cross flow of 0.1 mL/min. Channel flow was 0.2 mL/min. Fractions (0.6 or 0.8 mL) that were collected from the beginning of elution phase were stored at +4 °C or –20 °C.

At least three technical repetitions were analyzed per each input sample. In addition, various biological virus batches were used for the treatments. Buffer backgrounds were determined by analyzing control treatments, where the virus/NC was replaced with the corresponding volume of the buffer (Figure S2, Supplementary Materials). AF4 for non-treated, BHT-, and SDS-treated φ6 was performed in a 10 mM potassium phosphate buffer (pH 7.2) containing 1 mM MgCl_2_. NC was fractionated using a buffer that contained 10 mM potassium phosphate buffer (pH 7.2), 1 mM MgCl_2_, 0.1 mM CaCl_2_, and 150 mM NaCl. For the NC core, a 10 mM potassium phosphate buffer (pH 7.2), containing 1 mM MgCl_2_, 150 mM NaCl, and 20 mM EGTA, was used.

### 2.3. Analyses of Biological Activity, Purity and Yield

Protein concentrations were estimated from the A_280_ values (Eppendorf Photometer, Hamburg, Germany). The number of infectious viruses (plaque forming units, PFU) was determined by plaque assay on a lawn of *Pseudomonas syringae* HB10Y strain. Recoveries (%) of protein and infectious viruses were calculated from A_280_ values or PFU using the following formulas: (100% × A_280,AF4_/A_280,input_) or (100% × PFU_AF4_/PFU_input_). For NC that was purified to near homogeneity, 1 A_280_/mL equaled to ~0.13 mg of protein/mL.

Proteins were precipitated with 10% (*v*/*v*) trichloroacetic acid to assess the protein composition of collected AF4 fractions or the corresponding input samples. Resulting proteins were analyzed by SDS polyacrylamide gel electrophoresis (SDS-PAGE) using 16% polyacrylamide gels [40]. Separating gels were stained using Coomassie blue to detect proteins or with Sudan Black B to stain lipids. Stacking gels were stained with ethidium bromide (EtBr) to detect RNA. Alternatively, RNA was analyzed in 0.8% (*w*/*v*) agarose gels that were stained with EtBr. Stained gels were documented using ChemiDoc (Bio Rad, Hercules, CA, USA).

The functionality of the AF4-purified NC was studied by transcription assay [9,41]. Synthesis of plus-strands was carried out in 20 μL reactions by mixing equal volumes of the transcription buffer and NC mixture. The transcription buffer was prepared as 2× concentrate and it contained 100 mM Tris (pH 8.0), 100 mM NH_4_AC, 50 mM KCl, 10 mM dithiothreitol (DTT), 6 mM MnCl_2_, 2 mM MgCl_2_, 4 mM NTP mix, and 1 U/μL of RNase inhibitor (Fermentas, Waltham, MA, USA). The NC mixture contained 8.8 μL of the AF4 fraction, 0.2 μL 1 M Tris (pH 8.0), and 1 μL H_2_O. The final concentration of NaCl/KCl in transcription reactions was 120 mM. After incubation at 30 °C or on ice for 90 min, the reactions were stopped by adding a 2× U loading buffer [42] that contained 7% (wt/vol) EtBr. The reaction products (~10 μL of the transcription reaction) were heated at 50 °C for 5 min and analyzed in 0.8% (wt/vol) agarose gels.

The biological activity of the NC was assayed by infection of spheroplasts as described previously [43,44]. A receptorless φ6-resistant derivative of HB10Y, MP0.16, was used for their preparation. After mixing the spheroplasts (30 μL) with an equal volume of the NC2 input or the NC-containing AF4 fractions, the mixtures were incubated at RT for 50 min prior to diluting to 10^–2^–10^–6^ and plating on a lawn of HB10Y strain. As controls, the corresponding AF4 fractions were also directly titrated against HB10Y to confirm that the NC preparations contained no infectious viruses. The detection limit was 10^3^ PFU/mL.

## 3. Results and Discussion

### 3.1. BHT- and SDS-Treatment of φ6

**Figure 2:**
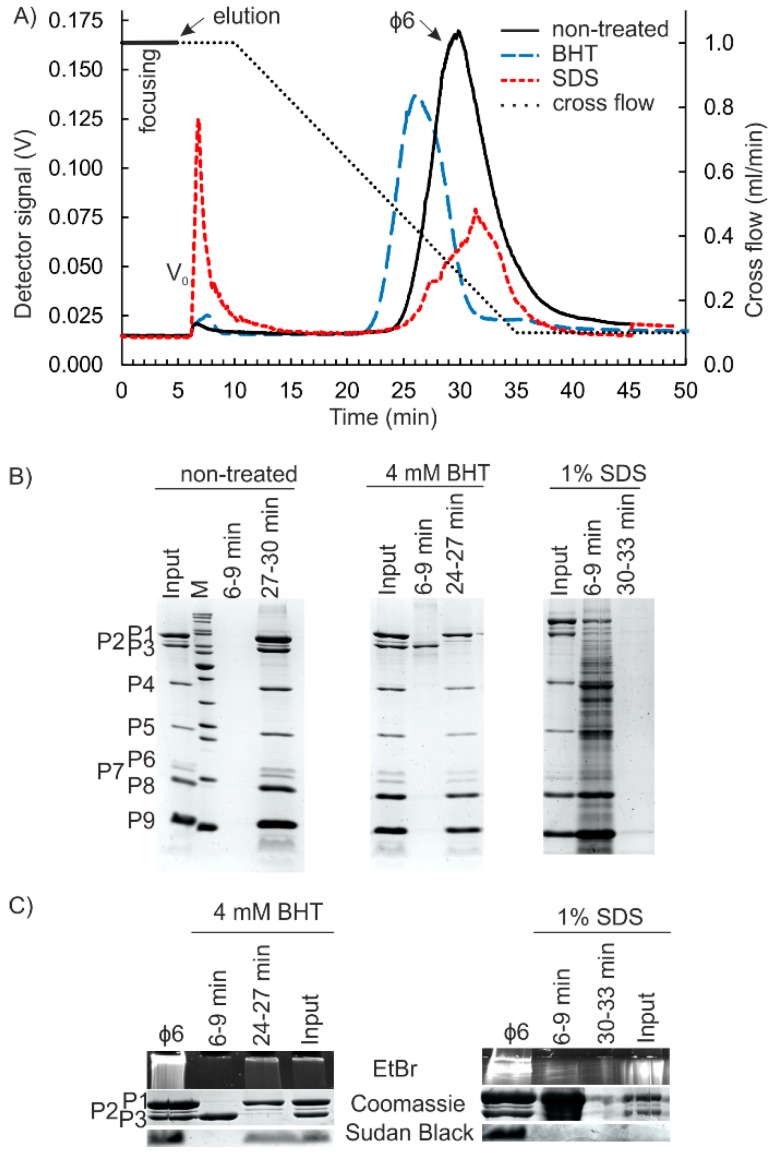
BHT- and SDS-treatment of φ6 virions. (**A**) AF4 fractograms for BHT-, SDS-, and non-treated φ6. Cross-flow elution gradient is shown with a dashed line (right y-axis). Detector signal intensity was measured at 260 nm in volts (V) (left y-axis). V_0_ is the void peak. (**B**) Protein content of the peak fractions was compared to the corresponding input samples. Equal volumes of fractions representing the peak maxima were analyzed. φ6 proteins are indicated on the left. M shows the migration pattern of Thermo Scientific PageRuler Unstained Protein Ladder #26614 (10 to 200 kDa). (**C**) Presence of lipids (Sudan Black B staining) and RNA (EtBr staining of the stacking gel) in the AF4 fractions and the corresponding input samples of BHT- and SDS-treated φ6. Coomassie stain for P1–P3 proteins is shown for comparison.

AF4 analysis of the non-treated φ6 particles resulted in elution of one major peak (Figure 2A). The retention times at the peak maxima varied from ~28 min to 29.5 min between experiments that were performed with different virus batches and on different dates (Figure S3A, Supplementary Materials). Our recent AF4-multi-angle light-scattering measurement (MALS) study showed that ultracentrifugation-based purification of φ6 yields virus specimen that has a relatively homogenous size distribution and little aggregates [5]. Virus-sized particles are generally well retained in the AF4 channel and elute at low cross-flow rates [5,38,39,45]. When compared to our previous study on φ6 that was performed with a thicker 350 μm spacer and resulted in virus elution at the end of the cross-flow gradient [5], here the thinner spacer promoted virus elution at higher cross-flow rates (peak maxima at a cross-flow rate of ~0.25 mL/min), providing improved resolution between monomeric viruses and putative larger sample components that are present if purification is performed with less purified inputs such as lysates or virus precipitates [5]. Analysis of the protein composition by SDS-PAGE (Figure 2B) and the number of infectious particles by plaque assay showed that φ6 eluted in the major peak. The average content of infectious φ6 and A_280_ units in the major peak were 73 ± 2% (*n* = 3) and 79 ± 7% (*n* = 6) of the input. The obtained yield and purity (specific infectivity value for the virus peak was ~9 × 10^11^ PFU/A_280_) were comparable to those previously reported [5]. Good recovery of prokaryotic viruses with various biophysical properties after AF4 purification has also been reported previously [38,39].

BHT-treatment strips P3 spikes from the φ6 envelope and causes a drastic decrease in infectivity, since the spikes are needed in host recognition [7]. The lipid envelope and the embedded proteins are unaffected [46]. Here, the BHT-treated input sample (1.8 × 10^9^ PFU/mL) had less than 1% of the infectivity of the non-treated control (9.5 × 10^11^ PFU/mL). Obtained AF4 fractograms showed an increase in the first peak intensity and a concomitant decrease in the second peak intensity for the BHT-treated sample when compared to the non-treated control (Figure 2A). BHT-treated viruses and non-treated viruses cannot be separated by rate-zonal centrifugation in sucrose gradient [7]. In AF4, the BHT-treatment advanced the elution of the major peak by ~3 min when compared to the non-treated virus (Figure 2A). This indicated a smaller hydrodynamic radius for the BHT-treated particles. P3 spikes of intact virions have been reported to extend ~2 nm from the surface of the viral lipid envelope [6].

Analysis of the protein content of AF4 fractions by SDS-PAGE verified that P3 eluted at the beginning of the cross-flow gradient in the first fractions and was thus successfully released by the BHT-treatment (Figure 2B). The used low-flow rates were optimized for the separation of virus-sized macromolecular complexes, whereas soluble proteins that are smaller than ~700 kDa were expected to elute as a single peak at the beginning of the cross-flow gradient [38,39]. Based on the A_280_ measurements, 62 ± 12% (*n* = 7) of the BHT-treated virus was recovered in the major peak that eluted at the end of the cross-flow gradient. Comparable ~60% values have been reported previously for the BHT-treated particles that were purified by rate-zonal and differential ultracentrifugation [44]. The first AF4 fraction for soluble proteins contained ~2.2 ± 0.3% (*n* = 7) of the input sample. Sudan Black B and EtBr staining verified that the particles in the main peak contained lipids as well as RNA, as expected (Figure 2C).

φ6 virions are completely disrupted by anionic detergents [47]. In our study, the plaque assay for SDS-treated virus was negative, indicating that the dissociation was complete. AF4 fractionation resulted in fractograms that showed a characteristically high-intensity peak at the beginning of elution that was absent in the non-treated control (Figure 2A). Part of this high intensity was due to a transient increase in pressure at the beginning of elution, which interfered with the UV measurement and was caused by SDS (Figure S2, Supplementary Materials). The second broad peak with low intensity was observed at the end of the cross-flow elution gradient (Figure 2A). Recoveries calculated based on the A_280_ units indicated that 59 ± 13% (*n* = 5) of the UV-absorbing input material eluted in the first peak containing soluble proteins, whereas the broad low-intensity peak contained the rest. SDS-PAGE analysis of the AF4 fractions showed that the majority of φ6 proteins were found in the first peak, whereas little protein was detected in the second peak (Figure 2A), indicating full dissociation of virions. A strong staining for RNA was observed in the stacking gel for the fractions that presented the slowly eluting second peak (Figure 2A). Therefore, we fractionated purified genomic RNA of φ6 to see how it elutes in AF4 (Figure S4A, Supplementary Materials). The obtained fractogram overlapped with that of the second peak of the SDS-treated φ6 (compare Figure 2A and Figure S4A, Supplementary Materials). Agarose gel analysis of the RNA content of the fractions indicated that the differently sized segments were not well separated, even though the intensities of the S, M, and L segments varied in the analyzed fractions (Figure S4B, Supplementary Materials). No lipids were detected in the SDS-treated input sample or in the AF4 fractions (Figure 2C).

### 3.2. Nucleocapsid (NC) Isolation

φ6 NC contains P1 (85 kDa), P2 (75 kDa), P4 (35 kDa), P7 (17 kDa), and P8 (16 kDa), as well as the three genomic dsRNA molecules. It is obtained in vitro from the infectious virions by extracting the lipid envelope and the associated proteins. This is classically achieved by non-ionic detergent treatment that is followed by subsequent purification using rate-zonal and differential ultracentrifugation [24,36]. We performed AF4 fractionation for NC1 and NC2 inputs (see Figure S1, Supplementary Materials) to study whether AF4 could be utilized for purification of functional NC. Plaque assay for the various NC1 and NC2 input batches yielded virus titers that were below 10^4^ PFU/mL, implying efficient envelope removal, since those of purified φ6 are usually ~10^13^ PFU/mL [5].

**Figure 3:**
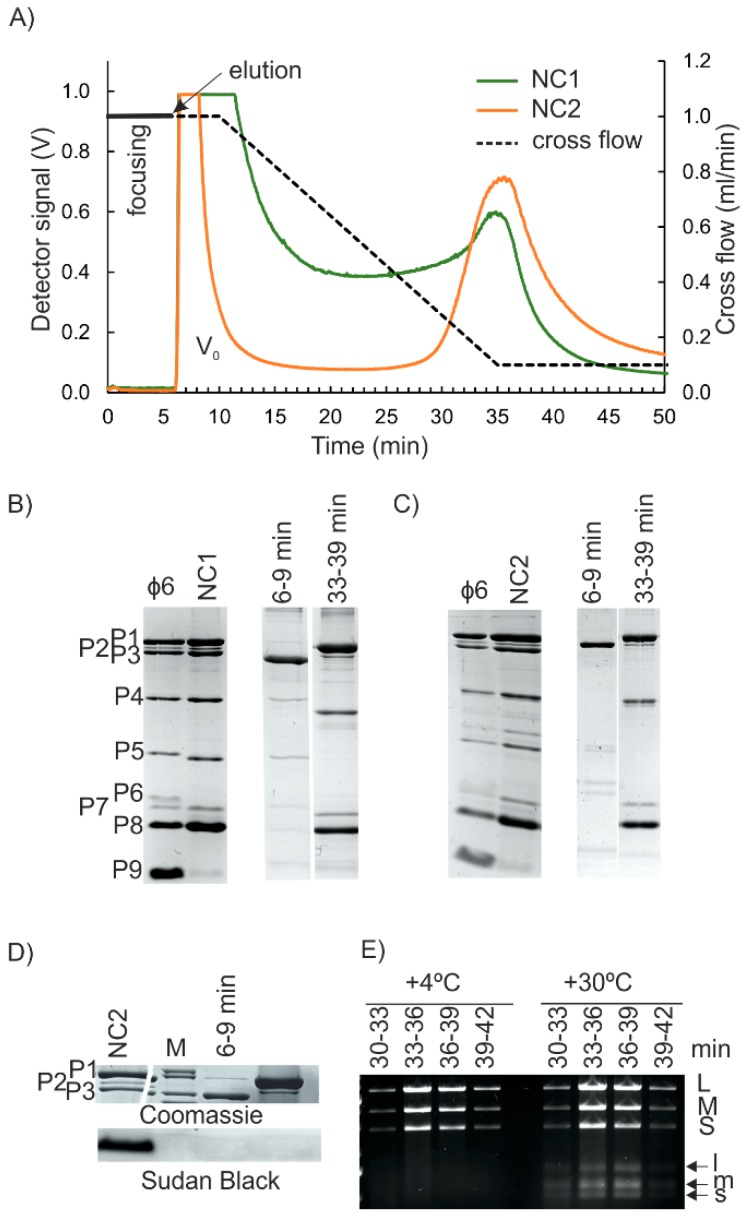
AF4 purification of φ6 NC. (**A**) AF4 fractograms for Triton X-114-extracted φ6. The cross-flow elution gradient is shown with a dashed line (right y-axis). Detector signal intensity was measured at 260 nm in volts (V) (left y-axis). V_0_ is the void peak. (**B**,**C**) Protein content of input samples and fractions at the peak maxima after Triton X-114 treatment (NC1 input) (**B**) or after subsequent purification in an 8% sucrose cushion (NC2 input) (**C**). Non-treated virus is shown as a control. Migration pattern φ6 proteins are indicated on the left (**C**). (**D**) Lipid staining (Sudan Black B) for AF4 fractions and the NC2 input sample. Coomassie stain for P1–P3 proteins is shown for comparison. (**E**) Transcription activity of the indicated AF4 fractions at +4 °C and at an optimal reaction temperature of +30 °C. Agarose gel electrophoresis analysis of the reaction products. Positions of genomic dsRNA molecules (L, M, S) and ssRNA transcription products (l, m, s) are indicated on the right.

First, AF4 purification of NC1 input (see Figure S1, Supplementary Materials), the water phase from the Triton X-114-treated virions was studied. AF4 analysis that was performed with the corresponding buffer control resulted in a high-intensity peak at the beginning of elution that was caused by the UV-absorbing properties of Triton X-114 and indicated that the water phase contained residual amounts of the detergent (Figure S2, Supplementary Materials). In the case of Triton X-114-treated virions, the first high-intensity peak was followed by a broad late-eluting peak (Figure 3A). As NC is a round structure with a smooth surface and a diameter of ~54–60 nm [6,17,18], we expected that it would have eluted earlier than the BHT- or non-treated viruses with larger diameters (compare Figure 2A and Figure 3A; see also Figure 1). AF4 fractionation of NC preparation that was purified to high homogeneity using anion exchange chromatography [19] resulted in similar high-retention and late-elution behavior (Figure S5, Supplementary Materials).

Approximately 30% of the φ6 protein mass is found on the membrane envelope [46] and ~50% is NC-associated [22]. The membrane-associated proteins include the major membrane protein P9 (9.5 kDa), as well as the minor proteins P6 (17.2 kDa), P10 (4.2 kDa), and P13 (7.7 kDa) [22,48]. Detergent treatment releases the spike protein P3 (69 kDa) and also P5 (24 kDa), since P3 is bound to the membrane via P6, and P5 resides between the membrane and NC [49]. Consequently, φ6 proteins P9, P10, P13, and P6 are mainly found in the Triton phase after detergent treatment, whereas P5 and P3 are predominantly in the water phase of NC preparations [22,23]. The low amount of P9 in the NC input samples indicated that it was efficiently trapped in the detergent phase, whereas AF4 fractionated P3, P5, and P6 into the first peak (Figure 3B). Due to the small sizes of P10 and P13, they could not be detected with the gel system used. Furthermore, if present, they were expected to pass through the 10 kDa RC membrane during AF4 separation. The well-retained second peak contained the expected ratios of protein components of φ6 NC: P1, P2, P4, P7, and P8 [44]. Based on A_280_ measurements, the NC-containing peak eluting from 29 min to 41 min contained 28 ± 1% (*n* = 4) and the first peak 32 ± 9% (*n* = 4) of the input sample, respectively. Comparable ~25 % recoveries have been previously reported for the NCs that were purified from Triton X-114 extractions by ultracentrifugation [44].

AF4 fractionation was repeated with NC2 input, whereas NC1 was further processed by a low-speed centrifugation in an 8% sucrose cushion prior to the AF4 (Figure S1, Supplementary Materials). The obtained fractograms were similar to those obtained for NC1 input (Figure 3A). SDS-PAGE analysis showed that P3, P5, and P6 were found in the first peak, whereas the second broad peak was enriched with the protein components of the NC (Figure 3C, and Figure S6A, Supplementary Materials). Sudan Black B staining indicated that even though some residual amount of lipids was present in the NC2 input, they could not be detected in the AF4-fractionated NC (Figure 3D). Based on A_280_ measurements, the average yield for NC was 39 ± 8% (*n* = 19), and thus was slightly higher than the yields obtained with the NC1 input. Depending on the amount of NC used, one AF4 fractionation yielded ~80–350 μg of NC. The first peak contained 6.3 ± 1.7% (*n* = 19) of the UV-absorbing components of the input.

Fractions that contained the NC proteins also included the three φ6 genomic dsRNA segments (Figure 3E, and Figure S6B, Supplementary Materials). The intensities of the dsRNA segments in the NC-containing AF4 fractions were similar (Figure 3E, and Figure S6B, Supplementary Materials), whereas fractions that were collected from AF4 analysis of purified dsRNA of φ6 showed differences in the amounts of S, M, and L segments (Figure S4B, Supplementary Materials). This indicates that the AF4-purified NCs are intact. This conclusion is supported by the finding that RNAse A treatment of NC input prior to the AF4 has little effect on the elution of the NC-containing peak or the protein and RNA content and the transcription activity (see below) of the NC-containing fractions (Figure S6C,D, Supplementary Materials). Cryo electron microscopy studies have shown that some NCs tend to lose their RNA content [18,19]. Thus, RNAse A treatment that is followed by separation of the RNAse A from NC (Figure S6D, Supplementary Materials) may be advantageous for some applications, where the free genomic RNA would be problematic.

Successfully purified φ6 NC has in vitro transcription activity [9,24,47]. It is mediated by a semi-conservative strand replacement mechanism, where the newly synthesized RNA strands replace the old ones, and the old replaced single-stranded (ss)RNA molecules start to accumulate [50,51]. We used the well-established in vitro transcription assay to test the functionality of the AF4-purified NC [9,41]. Fractions that contained the P1, P2, P4, and P7 proteins and genomic RNA segments characteristic for NC (Figure 3C,E, and Figure S6A,B, Supplementary Materials) were also transcriptionally active as indicated by the production of s, m, and l ssRNA molecules (Figure 3E, and Figure S6D, Supplementary Materials).

The biological integrity of the AF4-purified NCs was also tested by studying their capacity to infect host cells. As NC lacks the lipid envelope and associated proteins needed for normal infection that is initialized by membrane fusion between the virion and host membranes [23], the assay was performed using spheroplasts with partially disturbed outer membranes [43,44]. AF4 fractions that contained NC induced the formation of ~3 × 10^5^–~3 × 10^6^ infectious centers/mL (IC/mL) (Figure S7, Supplementary Materials), whereas no plaques were formed when the corresponding fractions were titrated against intact φ6 host cells. In summary, the results of this assay indicate that in addition to being transcription competent, AF4-purified NCs can initiate a productive viral replication cycle.

The high retention of NC in the AF4 channel was puzzling. Interaction of NC with the negatively charged RC could retard its elution. However, φ6 NC binds to anion exchange columns, indicating that it has negative surface charge [19]. Therefore, repulsion and enhanced elution would be more likely. Aggregation or the presence of substances that promote NC to equilibrate close to the accumulation wall could induce the late elution behavior as well. Re-fractionation of the NC-containing AF4 fractions resulted in the same retention times for fractions eluting from 33 min to 36 min, and from 36 min to 39 min, indicating that the fractions contained NC that was homogenous in size (Figure S5A, Supplementary Materials). The fact that the AF4-fractionated NC was biologically active (Figure 3E, and Figure S7, Supplementary Materials) and NC preparation that was purified to high homogeneity gave a similar fractogram (Figure S5B, Supplementary Materials) strongly suggested that there were no aggregates present and that the residual amounts of Triton X-114 did not cause the late elution of NC. AF4 fractionations for NC inputs were performed in the presence of 150 mM NaCl to stabilize the NC [24]. The increased ionic strength has been shown to affect the elution behavior of sample components that is specific for each component [39]. Here, the 150 mM NaCl concentration used had only a minute effect on the retention time of the virus peak (Figure S3B, Supplementary Materials). In general, elution at the end of the cross-flow gradient can result in potential contamination of the AF4-purified NC with other well-retained sample components. However, the contamination risk depends on the nature of the input sample. For instance, successful AF4 purification of φ6 and five other prokaryotic viruses from fractions eluting at the end of the cross-flow gradient did not compromise the purity or the integrity of the purified viruses [5,38,39]. Here, co-elution of the free genomic RNA of φ6 and NC could be solved by RNAse-treatment of the input sample (Figure 3, and Figures S3 and S4, Supplementary Materials). However, empty and functional RNA-containing NCs are the same size [18,19] and cannot be separated in AF4.

### 3.3. Isolation of the NC Core

**Figure 4:**
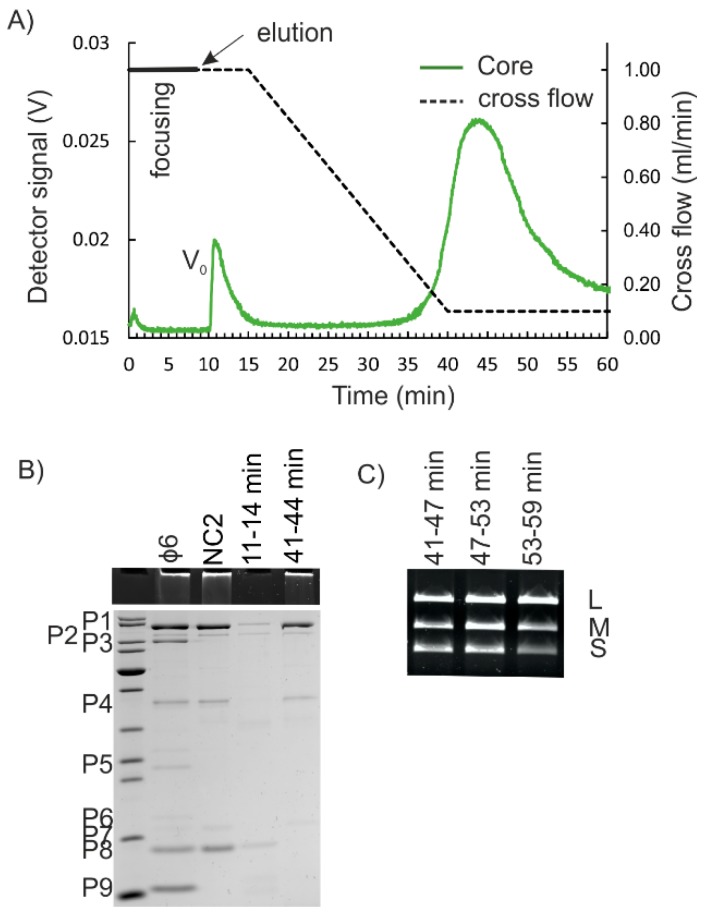
AF4 purification of φ6 NC core. (**A**) AF4 fractogram for NC that was treated with 25 mM EGTA to remove P8. NC was obtained from AF4 purification of NC2 input. The cross-flow elution gradient is shown with a dashed line (right y-axis). Detector signal intensity was measured at 260 nm in volts (V) (left y-axis). V_0_ is the void peak. (**B**) Protein content of input sample and AF4 fractions at the peak maxima. Presence of RNA was detected from the stacking gel using EtBr staining. M shows the migration pattern of Thermo Scientific PageRuler Unstained Protein Ladder #26614 (10 to 100 kDa shown). The mobility of the φ6 proteins is indicated on the left. (**C**) Agarose gel electrophoresis for RNA content of fractions representing the major peak. Positions of L, M, and S dsRNA molecules are indicated on the right.

Removal of the P8 layer from NC yields NC core particles. They are obtained in conditions that chelate Ca^2+^ ions and induce disassembly of the P8 shell [24,37] (Figure 1). However, the NC core particles are fragile and easily disrupted [17,37]. We next studied whether the purification of NC cores could be achieved using AF4. Two different approaches were tested for P8 removal. The NC2 input or the corresponding AF4-purified NC was treated with EGTA prior to the AF4 (see Materials and Methods). Obtained AF4 fractograms for the EGTA-treated NC were similar to those of non-treated NC inputs (compare Figure 3A and Figure 4A). NC core particles are spherical structures of ~50 nm in diameter with ~29 nm extensions made of P4 that protrude from the surface [17]. Thus, the overall hydrodynamic diameter of NC core is similar to that of NC. 

P8 accounts for ~30% to 50% of the total protein content of the NC [4]. Here, the yields for the NC core were 27.2 ± 9.9% (*n* = 4) of that of the input. Analysis of the protein patterns by SDS-PAGE verified that the NC core proteins P1, P2, P4, and P7 were present in the fractions that represented the second peak, whereas P8 was found in the first peak (Figure 4B). Fractions that contained the proteins characteristic for NC cores, contained the genomic RNA segments of φ6 (Figure 4C). The P8 layer is needed for penetration of the NC through the cytoplasmic membrane [44]. The fact that the AF4-purified NC core particles were incapable of infecting spheroplasts correlated with the SDS-PAGE results (Figure 4B) and showed that P8 was successfully removed.

## 4. Conclusions

AF4 has previously been successfully used to analyze the end products of the assembly and disassembly of murine polyoma virus virus-like particles [52,53]. It has also been used to evaluate the effect of temperature and excipients on influenza virus size distribution and aggregation [45,54]. We have previously utilized AF4 for purification of infectious viruses of various morphotypes and biochemistries [5,38,39]. Here, we showed that AF4 provided a rapid and efficient method to study conditions for the disassembly of the complex virions of phage φ6. Furthermore, we also showed that the controlled dissociation treatments, followed by AF4 purification, produced biologically active subassemblies. In conclusion, AF4 provides a rapid method to analyze the outcomes of many biochemical and biophysical treatments. It is applicable to viruses of various sizes, shapes and biochemical compositions. When coupled to light scattering detectors, data on the size can also be obtained.

## Supplementary Materials

The following supplementary material is available online at http://www.mdpi.com/1999-4915/10/11/579/s1, Figure S1: Preparation of NC inputs; Figure S2: Control treatments and buffer backgrounds; Figure S3: φ6 retention in AF4; Figure S4: φ6 RNA retention in AF4; Figure S5: Refractionation and AF4 fractionation of purified φ6 NC; Figure S6: Purification of φ6 NC by AF4; Figure S7: Capacity of AF4-purified φ6 NC to infect spheroplasts.
