# Supplementary material for: Controlled Disassembly and Purification of Functional Viral Subassemblies Using Asymmetrical Flow Field-Flow Fractionation (AF4)
Source: Viruses. 2018 Oct 23;10(11):579. doi: 10.3390/v10110579 (PMC6265779; doi:10.3390/v10110579)
Supplement: Supplementary file 1 [file viruses-10-00579-s001.zip › viruses-368410-supplementary.pdf]

Controlled disassembly and purification of functional viral subassemblies using asymmetrical flow field-flow fractionation (AF4).

Katri Eskelin and Minna M. Poranen

Supplementary Figures S1-S7.

Supplementary Fig. S1. Preparation of NC inputs.
Supplementary Fig. S2. Control treatments and buffer backgrounds.
Supplementary Fig. S3. ϕ6 retention in AF4.
Supplementary Fig. S4. ϕ6 RNA elutes at the end of cross-flow gradient.
Supplementary Fig. S5. Refractionation and AF4 fractionation of purified ϕ6 NC.
Supplementary Fig. S6. Purification of ϕ6 NC by AF4.
Supplementary Fig. 7. Capacity of AF4-purified ϕ6 NC to infect spheroplasts.

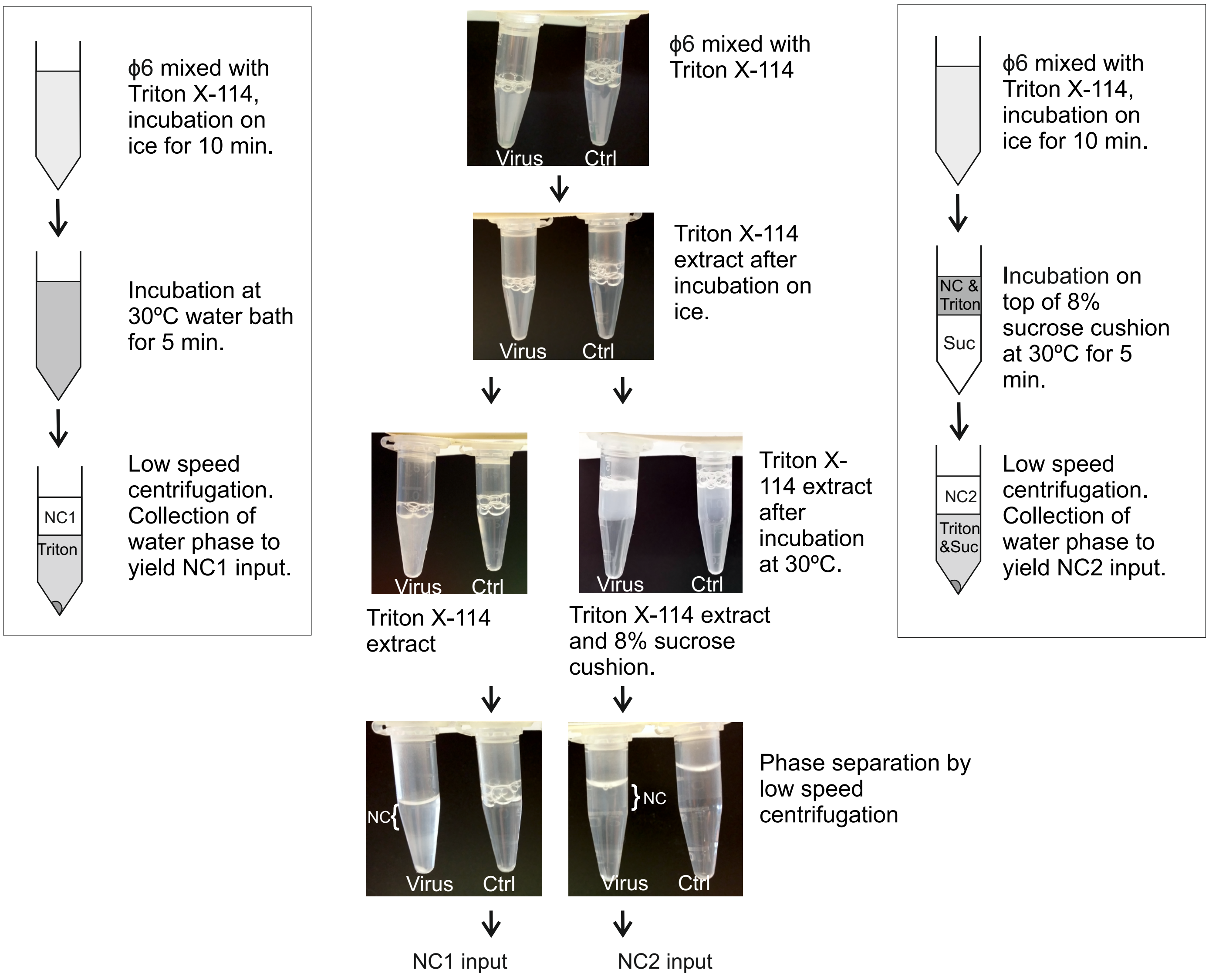

Supplementary Fig. S1. Preparation of NC inputs. Virus was first mixed with 1/3 volume of precondensed Triton X-114. In controls (Ctrl) the virus was replaced with buffer. After 10 min incubation on ice, the mixture was placed into a 30 ºC water bath for phase separation (left). Phase separation was enhanced by a low speed centrifugation, after which the NC-containing water phase was collected to result in NC1 input. Alternatively, after incubation on ice, the mixture was layered on top of an 8% sucrose cushion and incubated in the 30 ºC water bath (right). Phase separation was also obtained by a low speed centrifugation, after which the NC-containing water phase was collected to result in NC2 input.

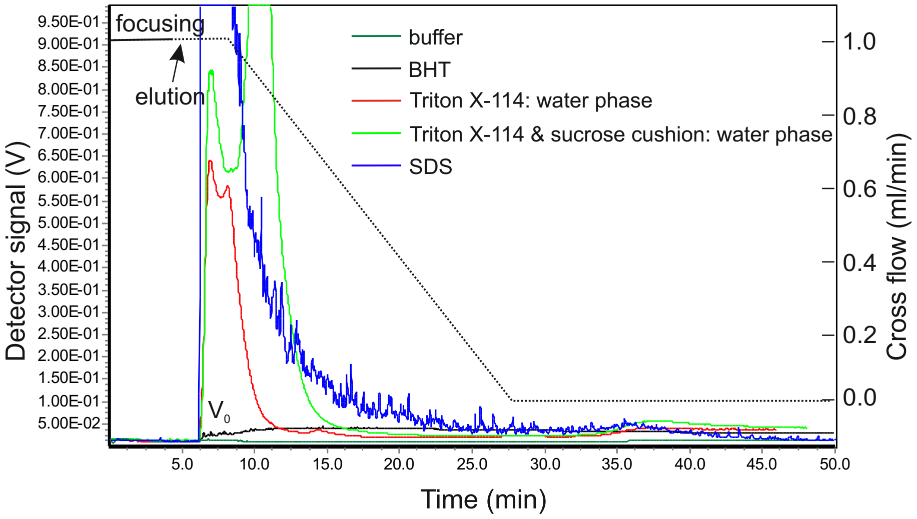

Supplementary Fig. S2. Control treatments and buffer backgrounds. Control treatments were performed without virus addition and resulting control samples were used for AF4 analysis. Signal intensity at 260 nm for SDS-, BHT-, and Triton X-114 treatments. For SDS-control, the high intensity peak at the beginning of elution was partially caused by a transient increase in the pressure that interfered UV measurement. The AF4 buffer used was 10 mM potassium phosphate (pH 7.2), 1 mM $MgCl_2$. Cross-flow elution gradient is shown with dashed line (right y-axis). Detector signal intensity was measured at 260 nm in volts (V) (left y-axis). V_0 is the void peak.

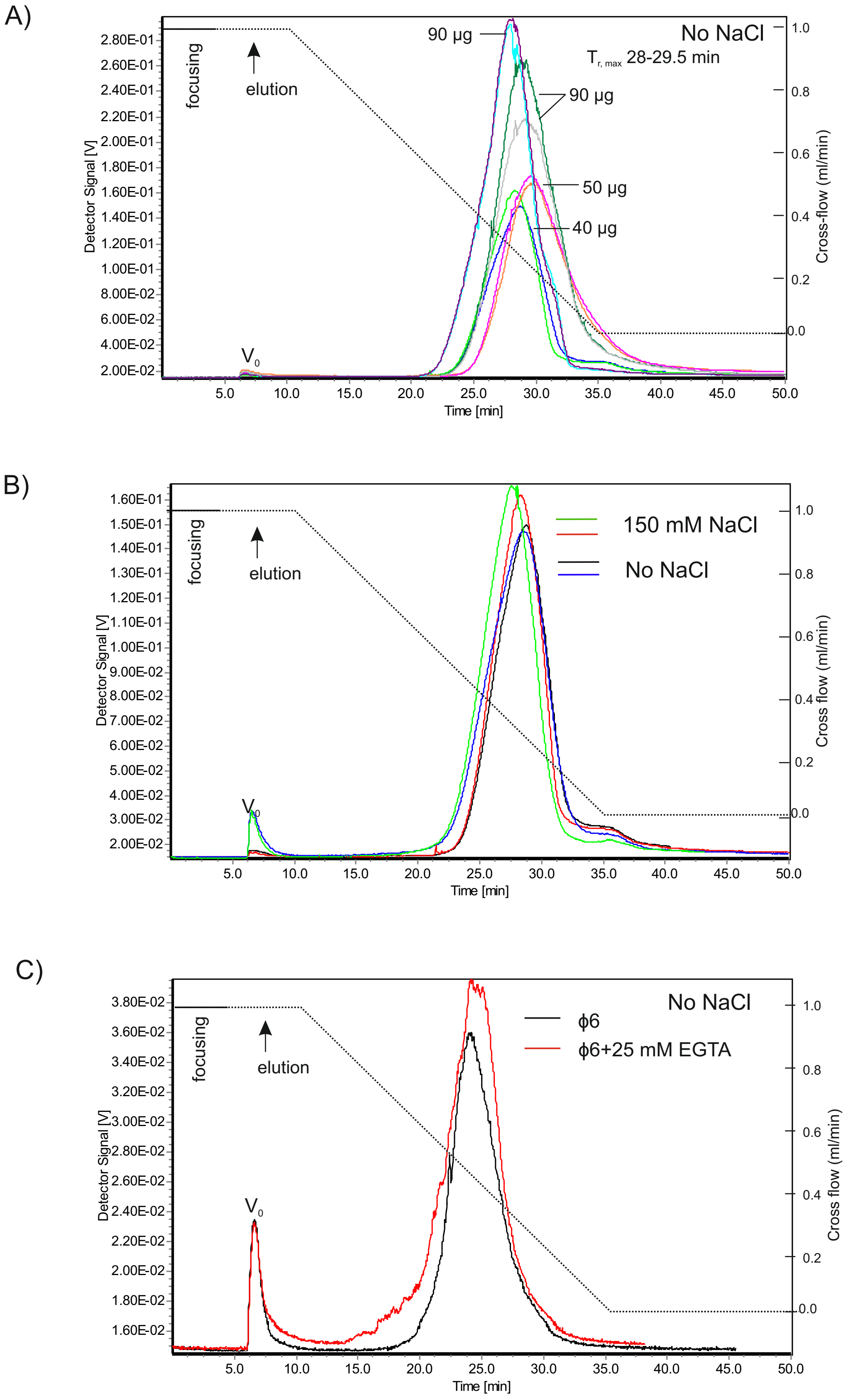

Supplementary Fig. S3. ϕ6 retention in AF4. A) Day-to-day and batch-to-batch variation in retention. Fractograms present data obtained for two virus batches that were analysed in duplicate on four different days. Injected virus amounts varied from ~40 to 90 µg. B) Effect of NaCl in ϕ6 retention. Analyzed virus amount was ~70 µg. C) Effect of 25 mM EGTA-treatment on elution of intact ϕ6. AF4 was performed using 20 mM potassium phosphate (pH 7.2), 1 mM $MgCl_2$ buffer that lacked or contained 150 mM NaCl. Cross-flow elution gradient is shown with a dashed line (right y-axis). Detector signal intensity was measured at 260 nm in volts (V) (left y-axis). V_0 is the void peak.

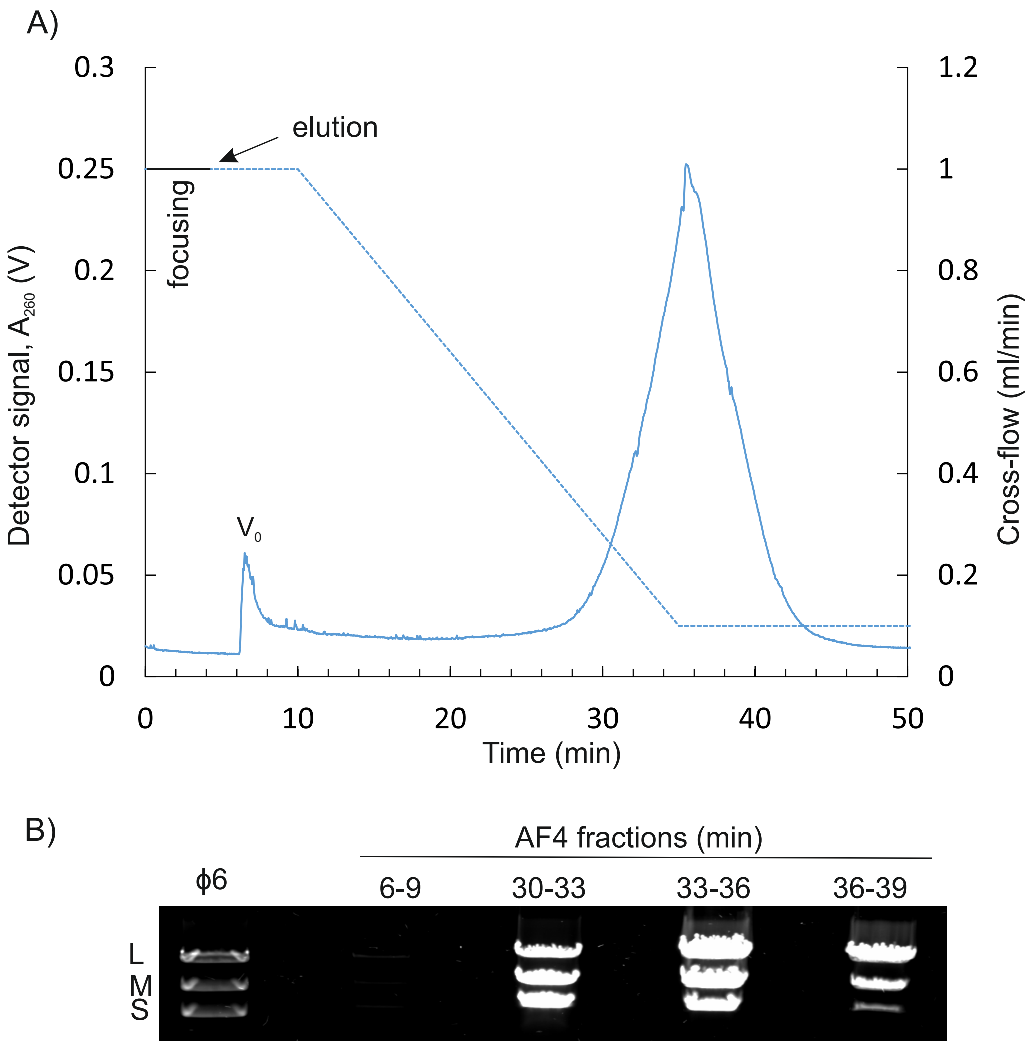

Supplementary Fig. S4. ϕ6 RNA elutes at the end of cross-flow gradient. A) Elution of purified genomic RNA of ϕ6 in AF4. UV detector signal at 260 nm is shown with a solid line in volts (V). Cross-flow gradient is shown with dashed line (right y-axis). V_0 is the void peak. B) Agarose gel electrophoresis for the RNA content of the major peak and ϕ6 input. Positions of genomic dsRNA molecules (L, M, S) are indicated on the left.

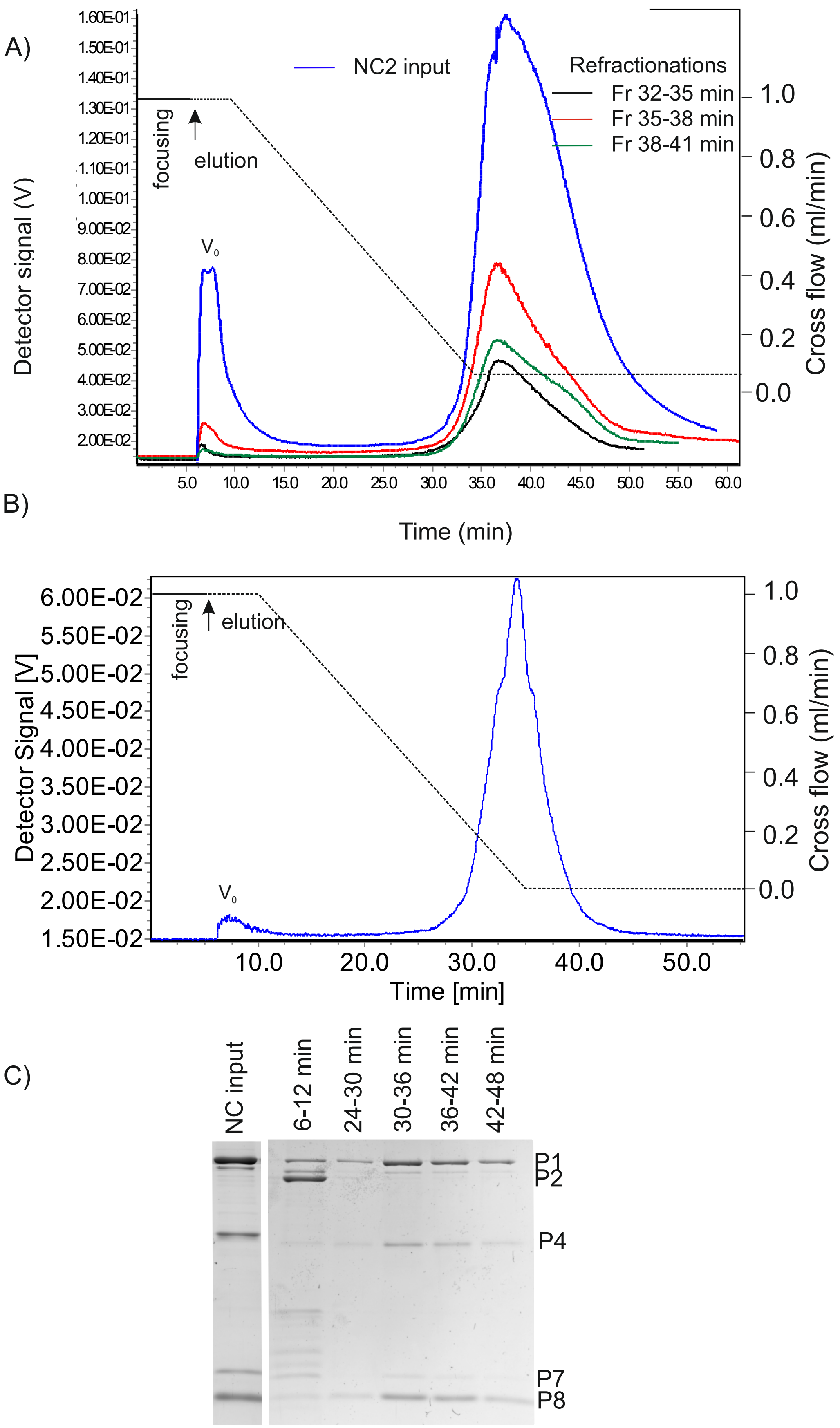

Supplementary Fig. S5. Refractionation and AF4 fractionation of purified ϕ6 NC. A) Refractionation of AF4 -purified NC2 input. Fractions representing the NC peak were re-injected to AF4 (á 500 µl) and compared to the input. Elution time for the analysed fractions are indicated in the figure. B) ϕ6 NC that had been pre-purified with anion exchange chromatography elutes at the end of the cross-flow gradient. UV signal that was measured in volts (V) at 260 nm is show with a solid line (left y-axis). Cross-flow gradient is shown with a dashed line (right y-axis). Focusing time was 5 min. V_0 is the void peak. B) Protein composition of the major peak. Protein components of ϕ6 NC are indicated on the right.

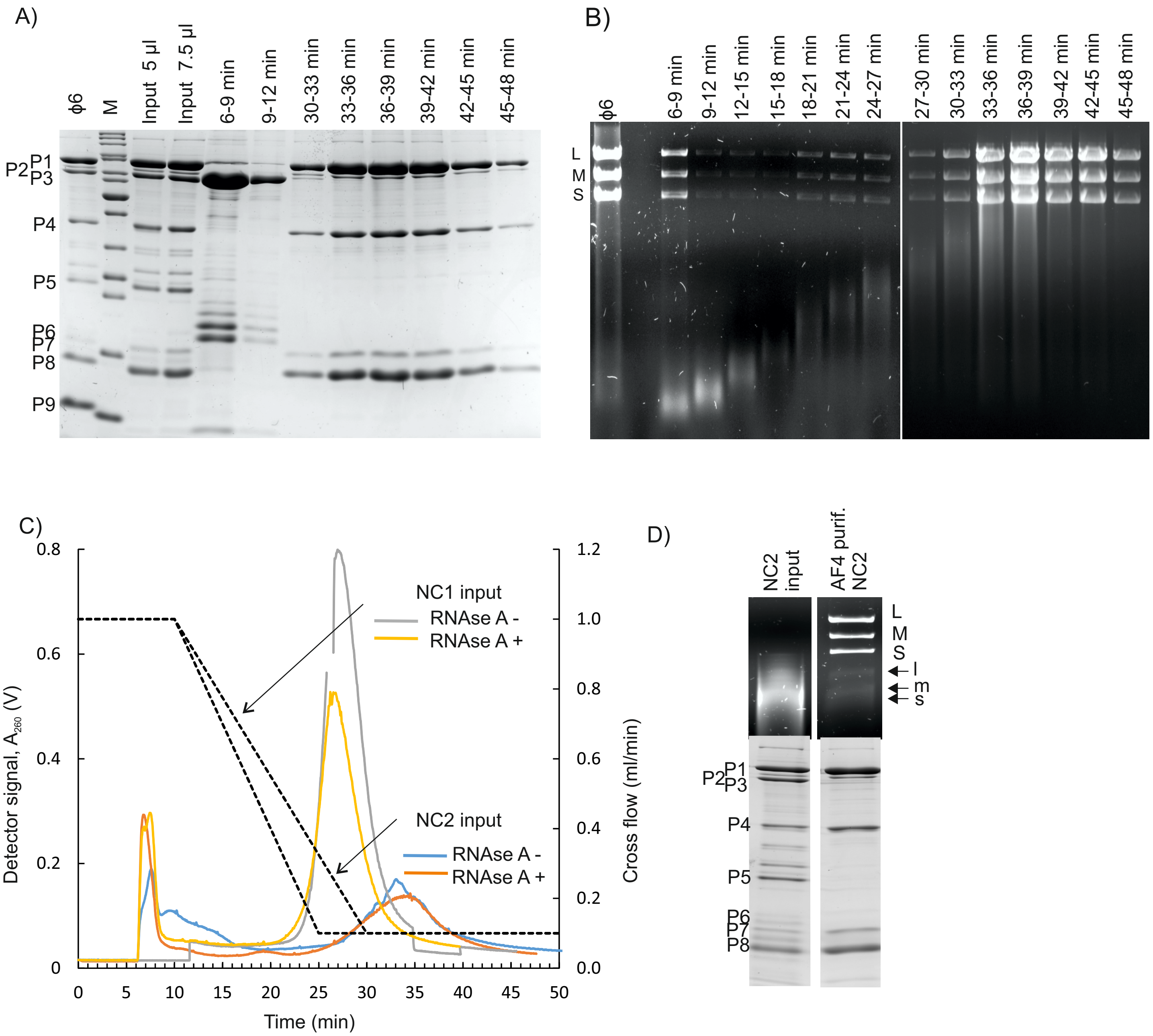

Supplementary Fig. S6. Purification of ϕ6 NC by AF4. A) Protein and B) RNA content of fractions from purifications where NC2 was used as input. See Fig. 3A for the corresponding fractogram. C) AF4 fractograms for RNAse A- and non-treated ϕ6 NC. RNAse A (50 µg) treatment was performed for 25 min at RT. Note that a shorter cross-flow gradient was applied for NC1 input (grey and yellow lines) that resulted in earlier elution of purified NC. D) Transcription assay for the RNAse A -treated NC2 input and the corresponding AF4 -purified NC reveals successful removal of RNAse A during AF4 (upper part), whereas protein analysis shows that ϕ6 envelope proteins were successfully removed from the AF4 -purified NC. ϕ6 proteins and RNA segments are indicated. M shows the migration pattern of Thermo Scientific PageRuler Unstained Protein Ladder #26614 (10 to 200 kDa).

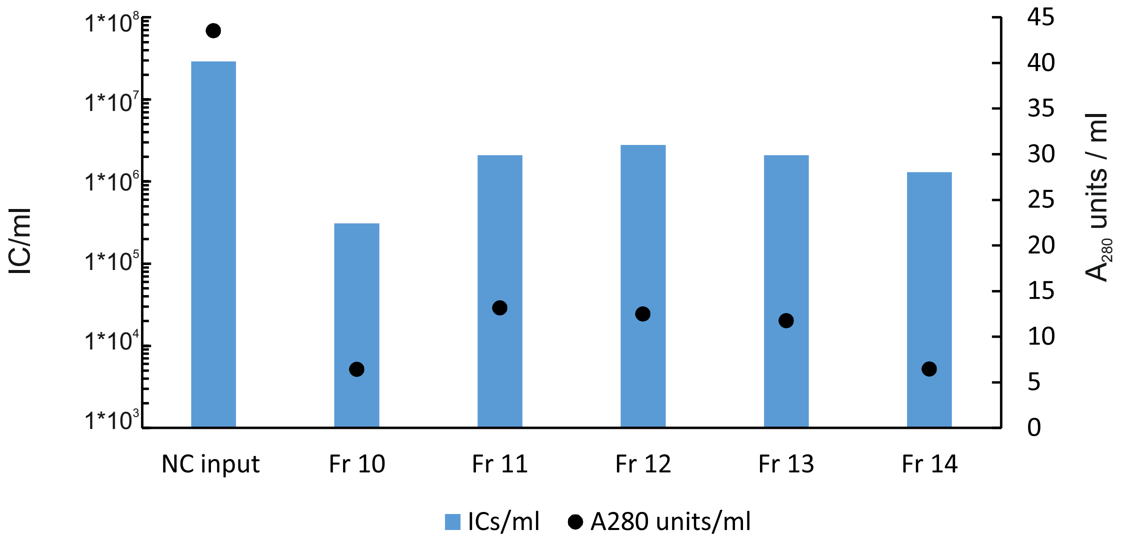

Supplementary Fig. 7. Capacity of AF4-purified ϕ6 NC to infect spheroplasts. As NC lacks the lipid envelope, spheroplasts were used to assay the capacity of the AF4 purified NC2 input to program the replication of ϕ6 and subsequent formation of infectious viruses (columns, left y-axis). Detection limit was $1*10^3$ IC/ml. Corresponding A_{280} units (dots) of the NC containing fractions are shown on the right y-axis.
